# Single-tooth replacement: factors affecting different prosthetic treatment modalities

**DOI:** 10.1186/1472-6831-11-34

**Published:** 2011-12-21

**Authors:** Firas A Al-Quran, Raed F Al-Ghalayini, Bashar N Al-Zu'bi

**Affiliations:** 1 P.O. Box 3030 IRBID 22110, Jordan University of Science and Technology (JUST)/Faculty of Dentistry, Irbid/Jordan; 2 P.O Box 27272, M28-135, University of Sharjah/Sharjah/United Arab Emirates; 3 Shemesani Dental Center, Shemesani/Amman, P.O. Box 8627 Amman 11121, Jordan; 4 College of Dentistry/Al-Jouf University, PO. B 2014 Al-Jouf - Skaka KSA

## Abstract

**Background:**

The choice between several treatment options for replacing a single missing tooth is influenced by clinical, dentist- and patient-immanent factors. This study aimed to determine the patient factors that would affect the treatment decision to replace a single missing tooth and to assess the satisfaction with several options.

**Method:**

200 volunteers involved (121 females and 79 males) divided into four groups, Group A: consisted of patients with conventional fixed partial dentures or patients with resin bonded fixed partial dentures. Group B: consisted of patients who received removable partial dentures while Group C: consisted of patients who received a single implant supported crown, and a control group D: consisted of patients who received no treatment. Data were collected using a questionnaire.

**Results:**

The highest percentage of males within groups (58%) was within the removable prostheses category. The majority of the subjects in the study reported that the main reason for replacing a missing tooth was for esthetic and function. Most important factor affecting the choice between treatment modalities was damaging the neighboring teeth. Pain, post operative sensitivity and dental phobia were important factors in choosing the prosthesis type and affected the control group patients not to have any treatment. The highest satisfaction percentage among groups studied was recorded for dental implants then FPD groups, while the least percentage were in both the control and RPD groups, for all aspects of function, esthetic and speech efficiency.

**Conclusions:**

The final choice between FPD, RPD and implant depended on several factors which affected the decision making; among these is cost and patients' awareness of the different treatment options.

## Background

Replacement of missing teeth has become one of the most important needs for patients attending clinics to restore esthetics and/or function. Many treatment modalities are available for replacing a single missing tooth; removable partial denture, fixed partial denture or dental implant. Each modality is a possible treatment option and has its own advantages and disadvantages [1]. There are several factors affecting the final treatment decision regarding the replacement of a missing tooth, these factors are case dependant. In many cases if more than one treatment option is possible, the definitive replacement depends on patient's decision/financial status or influenced by the patient's gender, age, public awareness and patient's knowledge. Therefore, it is mandatory to understand the patient's needs and demands to determine the kind of treatment that ensures the patient's satisfaction with the dental service. In many cases the cost of the treatment is considered as a major determinant and ahead of oral health status and patient preference. Pain and dental phobia are considered as important factors as well and they might affect the patient decision not to receive treatment at all [2,3]. Accessibility which highlight important differences between people. For example, a particular form of prosthetic treatment may be equally available to young and old patients, but the latter may find that the effort needed to seek out that treatment is just too great [4]. Treatment decisions cannot be performed depending on the basis of clinical examination or a dentist's opinion alone, but should be discussed in close consultation with patients [5]. In clinical decision making, dentists routinely choose between alternative treatments such as crown vs. amalgam/composite buildup; root canal treatment vs. extraction; fixed bridge vs. removable partial denture; and periodontal treatment vs. extraction. A number of clinical and patient factors can influence the dentist's choice of treatment in these situations. However, little is known about their relative importance. To address this issue, a list of clinical (e.g., periodontal status and caries rate) and patient (e.g., cost and patient preference) factors possibly influencing the choice of treatment was developed for each pair of services [6]. Decision making style was associated with service provision [7]. The term 'need' is commonly used to describe the amount of treatment that dentist's judge their patients ought to have, whilst 'demand' refers to the treatment requested by the patients themselves [8]. Most studies of prosthetic need and demand showed that the former is larger than the latter [9]. Other factors like the dentists, their particular skills, their accessibility to the public and the economic realities of the community in which they practise can affect the decision in choosing the treatment in addition to the attitudes of people towards different forms of treatment. These attitudes are influenced by such matters as education, personal finance, and cultural background [10].

It is objective to familiarize the patients with literature comparing success rates of fixed partial dentures, single-tooth implant restorations and a removable partial denture or techniques used in the replacement of single missing tooth. Some authors concentrated on clinical parameters when choosing different treatment options. Salinas *et al *[11] reported that the choice to replace a single missing tooth depends on the primary decision which is restorability of the tooth. The present attempt to define the concept patient satisfaction and to hypothesize some of its determinants can be regarded as the first step in building a theory of patient satisfaction. Hebel *et al *[4] discussed the advantages and disadvantages of different treatment modalities for restoring a single-tooth considering only the clinical situation without giving any importance to the patient's selection.

Despite the widespread concern in health care literature with patients' satisfaction, there has been no clear definition of that theory or the systematic consideration of its determinants and consequences. The replacement of a missing tooth by any of the prosthesis modalities occupies a major portion of the average restorative and prosthodontic practice. Treatment options keep changing due to continuous development [12].

Several options are currently available to address the challenge of restoring a single-tooth. To select the most appropriate treatment option for each patient, every case should be evaluated and all available options should be reviewed [13].

This study aimed to first, to analyse the factors that would affect the choice between different treatment modalities for replacement of a single missing tooth and second, to evaluate patients' satisfaction with the prosthodontic treatment they received.

## Methods

This study involved 200 participants who had only one missing tooth from anterior incisors to the second molars. 150 of those volunteers have been successfully treated and received their prosthesis at least one year before the study and the remaining 50 volunteers were control group and received no treatment. Participants were divided into four groups; each group consisted of 50 patients. Group A: consisted of patients who received conventional fixed partial dentures (FPDG) or patients with resin bonded fixed partial dentures (RBFPD). Group B: consisted of patients who received removable partial dentures (RPDG). While Group C: consisted of patients who received a single implant supported crown to replace their missing tooth (IG). Control group D: consisted of patients who received no treatment (CG). This study was approved by research committee at the Faculty of Dentistry and Faculty of Research at Jordan University of Science and Technology and the ethical approval committee at the university level.

Patients with special needs or mentally retarded and younger than 18 years old were not included in this study. Patients with two or more adjacent missing teeth and patients with edentulous spaces at the third molar area were excluded.

Patients were examined and if they have any complications with the prosthesis like signs of inflammation, they were excluded. Also repaired cases and crown fractures were considered complications.

The questionnaire was completed by patients. The questionnaire included 57 items that provided information regarding the patient's age, gender, martial status, education, job, monthly income, accommodation, medical and dental history, smoking habits and prosthetic rehabilitation. It also provided information concerning a patient's prosthetic knowledge, prosthesis kind, causes of tooth loss, factors affecting the choice of treatments, their prosthetic needs, source of information, their views regarding prosthetic rehabilitation and their overall satisfaction with their current prosthesis or situation esthetically, functionally and speech efficiency.

Each patient received a consent, which included a written explanation of the nature of the assessment to be undertaken.

An initial selection of the participants was made with emphasis on exclusion and inclusion criteria. Patients who did not meet inclusion criteria were referred for further treatment.

Collected data were statistically analyzed using Statistical Package for Social Sciences (SPSS, version 11.5). Data were described using means, standard deviations, and frequency distribution when appropriate. One way ANOVA was used to compare means of continuous variables between groupings variables, Post Hoc Multiple Comparisons were conducted after ANOVA. Chi-square test was used for data analysis where appropriate. P-values were calculated, if less than 0.05 was considered statistically significant.

## Results

The age of subjects in this study ranged between 19-67 years in all groups, with a mean age of 43.6 ± 10.4 (median 45). The 200 patients into this study were 121 female and 79 male. The baseline characterstics for all groups regarding gender, age, marital status, educational level and monthly income are shown in table 1.

**Table 1:** Baseline comparability of the treatment in socio-demographic factors between groups

Variables	*Fixed*		*Removable*		*Implant*		*Control*		*Total*	
	*N*	*(%)*	*N*	*(%)*	*N*	*(%)*	*N*	*(%)*	*N*	*(%)*
**Gender**										
Male	12	(24)	29	(58)	21	(42)	17	(34)	79	(39.5)
Female	38	(76)	21	(42)	29	(58)	33	(66)	121	(60.5)
*P- Value Vs control*	0.2705		0.0161		0.4099					
**Age**										
< 40	19	(38)	13	(26)	15	30)	23	(46)	70	(35)
40-50	14	(28)	22	(44)	18	(36)	13	(26)	67	(33.5)
> 50	17	(34)	15	(30)	17	(34)	14	(28)	63	(31.5)
*P- Value Vs control*	0.702		0.077		0.249					
Mean	42.9		46.4		44.7		39.4		43.6	
Range	(22-60)		(19-67)		(25-60)		(20-60)		(19-67)	
**Marital status**										
Single	14	(28)	9	(18)	18	(36)	17	(34)	58	(29)
Married	36	(72)	41	(82)	32	(64)	33	(66)	142	(71)
*P- Value Vs control*	0.5166		0.0682		0.8339					
**Education**										
< High School	21	(42)	23	(46)	23	(46)	36	(72)	103	(51.5)
> High School	29	(58)	27	(54)	27	(54)	14	(28)	97	(48.5)
*P- Value Vs control*	0.0024		0.0082		0.0082					
**Monthly Income**										
300 JD	40	(80)	38	(76)	20	(40)	42	(84)	140	(70)
> 300 JD	10	(20)	12	(24)	30	(60)	8	(16)	60	(30)
*P- Value Vs control*	0.6027		0.3173		< 0.0001					

All four groups were equivalent on age with no staistical differences and the same thing regarding the marital status.

The results showed that only 8% of all subjects in the current study did not go to school or did not finish their school education, while subjects with high school education were about 48.5%. Although higher proportion of subjects with prosthesis belongs to high school education group, there was significant difference among prosthesis type categories within this group.

The difference between FPD and RPD when compared with control groups regarding monthly income was not significant. However, the monthly income of patients in the implant group was higher than the income of the controls (*p*-value: < 0.0001).

In this study the location of missing tooth and its distribution in the four studies groups weather in aesthetic zone area or more towards posterior area is almost match able in all groups as shown in table 2 and this should exclude the differences between the groups regarding the location of the missing tooth.

**Table 2:** Location of the missing tooth.

	*Incisor* *N*	*Cuspid* *N*	*Premolar* *N*	*Molar* *N*	*Total* *N*
***Group A***	9	3	25	13	50
***Group B***	8	3	23	16	50
***Group C***	8	1	32	9	50
***Group D***	6	2	29	13	50
***Total***	31	9	109	51	200

Reasons for replacing the missing tooth in the patients of this study are summarized in table 3. Statistically significant differences were found between all groups (FPD, RPD and implant) when compared with controls regarding effect of missing tooth on patients relation with other people (P-value: 0.0007), (P-value: 0.0001) and (P-value: 0.0027), respectively. Also of all participants, 43.5% realized attention of others to their missing tooth.

**Table 3:** Reasons for replacing the missing tooth compared with control group who received no replacement.

	Fixed		Removable		Implant		Control		Total	
	N	%	N	%	N	%	N	%	N	%
**Attention of others**										
Yes	22	(44)	34	(68)	20	(40)	11	(22)	87	(43.5)
No	28	(56)	16	(32)	30	(60)	39	(78)	113	(58.5)
*P- Value Vs control*	0.0193		< 0.0001		0.0517					
**Esthetic Reason**										
Yes	47	(94)	44	(88)	38	(76)	4	(8)	133	(66.5)
No	3	(6)	6	(12)	12	(24)	46	(92)	67	(33.5)
*P- Value Vs control*	< 0.0001		< 0.0001		< 0.0001					
**Functional Reason**										
Yes	47	(94)	45	(90)	46 (92)		3	(6)	141	(70.5)
No	3	(6)	5	(10)	4	(8)	47	(94)	59	(29.5)
*P- Value Vs control*	< 0.0001		< 0.0001		< 0.0001					
**Replacement will do periodontal trauma**										
Yes	2	(4)	1	(2)	1	(2)	9	(18)	13	(6.5)
No	48	(96)	49	(98)	49	(98)	41	(82)	187	(93.5)
*P- Value Vs control*	0.0253		0.0076		0.0076					
**Missing tooth should be replaced**										
Yes	48	(96)	49	(98)	50	(100)	42	(84)	189	(94.5)
No	2	(4)	1	(2)	0	(0)	8	(16)	11	(5.5)
*P- Value Vs control*	0.0455		0.0144		0.0032					
**Bad Oral hygiene**										
Yes	28	(56)	24	(48)	32	(64)	29	(58)	113	(56.5)
No	22	(44)	26	(52)	18	(36)	21	(42)	87	(43.5)
*P- Value Vs control*	0.8399		0.3164		0.5385					

By excluding the controls, 60% believed that their missing tooth would affect their relation with others. Of this category 70% belonged to removable group then followed by 42% and 38% for the fixed and implant groups, respectively.

Regarding awareness of the dental prosthesis types, 60% had a good knowledge about the fixed prosthesis, compared to 47.5% about removable partial dentures and 57% for the dental implant. It was reported that 94% of implant group had a good background regarding implant therapy. On the other hand, 34% of the FPD and 72% of RPD groups had no background about dental implant.

Patients' satisfaction with their current prosthesis esthetically and functionally is shown in tables 4 and 5.

**Table 4:** Patient's current prostheses satisfaction esthetically.

Esthetically	Fixed		Removable		Implant		Control		Total	
	N	%	N	%	N	%	N	%	N	%
*Strongly satisfied*	13	(26)	10	(20)	46	(92)	3	(6)	72	(36)
*Satisfied*	27	(54)	15	(30)	4	(8)	17	(34)	63	(31.5)
*Neutral*	9	(18)	12	(24)	0	(0)	10	(20)	31	(15.5)
*Dissatisfied*	1	(2)	10	(20)	0	(0)	19	(38)	30	(15)
*Strongly dissatisfied*	0	(0)	3	(6)	0	(0)	1	(2)	4	(2)

**Table 5:** Patient's current prostheses satisfaction functionally.

Functionally	Fixed		Removable		Implant		Control		Total	
	N	%	N	%	N	%	N	%	N	%
Strongly satisfied	16	(32)	10	(20)	41	(82)	4	(8)	71	(35.5)
Satisfied	19	(38)	11	(22)	4	(8)	10	(20)	44	(22)
Neutral	12	(24)	12	(24)	5	(10)	11	(22)	40	(20)
Dissatisfied	3	(6)	14	(28)	0	(0)	25	(50)	42	(21)
Strongly dissatisfied	0	(0)	3	(6)	0	(0)	0	(0)	3	(1.5)

In all groups, 44% of subjects were strongly satisfied with their current prosthesis regarding their speech efficiency, 38.5% satisfied only, 9.5% neutral, 6.5% dissatisfied and only 1.5% was strongly dissatisfied (Table 6).

**Table 6:** Speech efficiency satisfactions regarding patient's current prostheses or situation.

Speech Efficiency	Fixed		Removable		Implant		Control		Total	
	N	%	N	%	N	%	N	%	N	%
Strongly satisfied	22	(44)	6	(12)	46	(92)	14	(28)	88	(44)
Satisfied	24	(48)	23	(46)	4	(8)	26	(52)	77	(38.5)
Neutral	4	(8)	8	(16)	0	(0)	7	(14)	19	(9.5)
Dissatisfied	0	(0)	10	(20)	0	(0)	3	(6)	13	(6.5)
Strongly dissatisfied	0	(0)	3	(6)	0	(0)	0	(0)	3	(1.5)

In addition, 67% of the study population believed that FPD treatment would not adversely affect neighboring teeth; 84% from FPD category, 56%, 82% and 46% from RPD, control and implant categories, respectively.

Of all participants, 83.5% believed in fixed partial denture while 81% believed in dental implants.

Regarding the factors that affected treatment choice, damage to neighboring tooth was one of the most important factors when choosing between different prosthesis types (40%) followed by pain and duration of the treatment, while cost of treatment was an important factor by only 27.5% of all participants as shown in table 7.

**Table 7:** Factors Affecting Treatment Option In Relation To Prosthesis Type.

	Fixed		Removable		Implant		Control		Total	
	N	%	N	%	N	%	N	%	N	%
**Cost**	8	(16)	17	(34)	2	(4)	28	(56)	55	(27.5)
**Pain and suffer**	18	(36)	17	(34)	20	(40)	22	(44)	77	(38.5)
**Surgery**	4	(8)	9	(18)	7	(14)	16	(32)	36	(18)
**Duration**	25	(50)	33	(66)	12	(24)	7	(14)	77	(38.5)
**Neighboring teeth**	17	(34)	18	(36)	28	(56)	17	(34)	80	(40)
**Phobia**	14	(28)	11	(22)	13	(26)	26	(52)	64	((32)

## Discussion

This study aimed at assessing factors that would affect the choice of different treatment modalities for the replacement of single missing tooth. On one hand, gender was found to be a patient factor that might influence treatment options. It was found that when females chose to replace the missing tooth, they usually favor fixed or implant treatment option more than removable treatment modality and this could be due to the fact that females are more apprehensive about their appearance and removable prosthesis makes them more conscious

On the other hand level of education played role in the choice whether to seek treatment or not. 72% of the subjects who were in the control group where less than high school education. That means the level of education could affect the patient's awareness regarding the importance of tooth replacement.

Of no surprise, 60% of patients with higher income chose the implant treatment option. Therefore, persons of low socioeconomic status tend to seek low cost treatment.

When patients where asked about factors affecting their choice of treatment modality overall, damage of adjacent teeth was the highest reported factor (40%) followed by duration of treatment (38.5%) and pain and suffer of the procedure (38.5). When looking at each treatment modality, duration was the highest deciding factor reported for both fixed and removable groups; 50%, 66% respectively. Long time needed for treatment by implant was not a major disadvantage according to the majority of our subjects. This was in agreement with Bragger *et al *[14] finding. They reported that total treatment time for FPDs and implant was similar, but implant required more visits than FPDs and RPDs. For implant group, damage to neighboring teeth was highest reported deciding factor 56%. As for control group, cost played the most deciding factor (56%), followed by dental phobia (52%). Hastreiter and Jiang [15] who reported that dental implants can provide various clinical and quality of life advantage compared to FPD and RPD, but it is more expensive than other principal single-tooth replacement alternatives. They stated that the average initial cost of a single-tooth replacement by implants (including surgical procedure and crown construction) is on average 35% more expensive than FPD but more expensive than RPD, and 105% more costly than root canal treatment with crown. A study by Tepper *et al *[16] reported that the cost was one of the most important factors for choosing dental replacement especially for implant treatment option. A common idea in restorative dentistry is to use a fixed prosthesis whenever possible. Rarely does a patient desire or accept a removable partial prosthesis as a substitute for a single missing tooth especially anterior tooth [17]. The usual indication for the removable option is economics. However, it still represents the easiest temporary treatment modality during submerged implant healing period. In this study only 34% of subjects preferred RPD because they are less costly than other treatment option which means that most of the subjects from RPD group in this study reported that they were not affected by cost. In this study only minority of subject's prefered RPD and their prefernce to this treatment option was that, it needs less time than other treatment options. Additionally Hebel *et al *[4] who reported in their study that one of the FPD advantages is that, it is completed in relatively short time, making duration of FPD treatment a deciding factor which is in agreement with what is found in our study. Kvale *et al *[3] did a meta-analytic and systematic quantitative approach to examine the effects of behavioural interventions for dental phobia. They found positive changes in 36 of the 38 studies and no changes in two. Our study showed that Dental phobia was reported by 32% of the study population as a factor affecting their treatment choice, most of these patients belong to control group.

From all treatment modalities RPD had the highest rate of dissatisfaction (26%) when compared with others. Zlataric *et al *[18] did a survey about treatment outcomes with removable partial dentures. They found that majority of the patients with RPDs were satisfied with the prosthesis and this might by explained by the fact that all of our participants have RPD replacing single missing tooth compared to wide range of RPDs in Zalatric *et al *study. Dissatisfaction was related to mastication, esthetics, number of missing teeth and maintenance of oral hygiene.

Patients as well as dentists' preferences regarding treatment options depend on several factors such as rejection to surgical procedures, treatment duration, cost, conditions of adjacent teeth or dental phobia. For a true economic evaluation, cost and benefits of different therapies are usually compared. The clinical outcome (benefit) in this study was 'single-tooth replacement'. Additional research is needed to assess lifetime costs that include initial and maintenance costs, and future replacement costs associated with various alternatives. Besides, most of the reported studies compared FPD cost with other modalities. A multivariate analysis by Arnbjerg *et al *reported that satisfaction with previous dental care depends primarily on three factors; treatment by the dentist of choice, chewing ability and satisfaction with their own dental conditions [19]. Our result showed that most of the subjects reported that missing tooth should be replaced for both esthetic and functional reasons.

As in any study, this investigation has its limitations. Being a case-control study, neither prevalence nor incidence can be calculated. As seen, majority of subjects were working with low income and low education level. Aditinally most of females in the studied sample were housewives or non-workers who were able to attend dental teaching center during its working time, whereas a high percentage of working males and females could not.

Many factors must be considered when chosing between different treatment options for the replacement of a single-tooth, often the bias of the dentist plays a role rather than objective appraisal of the treatment options.

Patient awarness of the advanteges and disadvanteges of different treatment modailities is very important for decion making, therefore there are many factors make single-tooth replacement one of the most challenging restorations in dentistry. As a result, for years patients were advised to place their desires aside and accept the limitations of a fixed partial denture. However, in light of the present technology, the major reasons for suggesting the fixed partial denture are its clinical ease and reduced time and cost. If this concept was expanded, extractions would replace endodontic treatment and removable partial dentures would replace fixed partial dentures or implant. Dentures could even replace orthodontics. The primary reason to suggest or perform a treatment should not merely be related to the cost, time, or difficulty to perform the procedure, but lays in the best possible long-term solution for each individual patient.

In reality, all treatment options offered advantages but also some disadvantages. The FPD restores three units; the single crown on an implant will just replace one tooth. With the implant reconstruction, no abutment teeth have to be prepared avoiding the risk for additional endodontic treatment, discomfort because of hypersensitivity, difficult access for plaque control, etc.

## Conclusions

In conclusion, among the multiple factors affected patients decision to the final treatment modality the replacement of single missing tooth single missing tooth, damage to the neighboring teeth and cost were most important. The highest satisfaction with aesthetic and function was in the implant group and the least in RPD group. The level of education and patients awareness towards different treatment modalities to replace single missing tooth have significant effect on the treatment choice. Attention of others to the patients' missing tooth and that might affect their relationship with others were important factors in all groups when compared with control group.

## Competing interests

The authors declare that they have no competing interests.

## Authors' contributions

FQ: principal research investigator, designed the study, set the questionnaire for the study in assistants with the other researchers, coordination, performed statistical analysis and editing the manuscript. RG: participated in the designing of the questionnaire, collected data, and related literature. BZ: Participated in the designing of the study and questionnaire, helped in data collection, analysis and writing up. Final manuscript is approved by all authors.

## Pre-publication history

The pre-publication history for this paper can be accessed here:

http://www.biomedcentral.com/1472-6831/11/34/prepub
